# A Capitation Grant Fallacy

**Published:** 1920-08-28

**Authors:** 


### The Hospital

The Workers' Newspaper of Administrative Medicine and Institutional Life, Administration, National Insurance and Health.

No. 1786, Vol. LXVIII. SATURDAY, AUGUST 28, 1920. PRICE TWOPENCE

### A CAPITATION GRANT FALLACY.

During the war the voluntary hospitals made themselves useful to the Government by receiving sick and wounded Service patients, and in return the Government made a grant to the hospitals on the basis of a capitation rate. The transaction was never intended to be on business lines and, as a temporary measure, skilled examination of the position from an accountancy point of view was not carried out. Obviously the sum paid by the War Office and the Admiralty—namely, 4s. and later 4s. 9d. a day—did not represent the total cost of the patient under all headings. The voluntary hospitals were pleased to render this patriotic service, and it is probable that they did not lose by it, for it no doubt brought them many new sympathisers and supporters.

When, however, the capitation grant principle is adopted by the voluntary hospitals as applied to patients for whose subsistence and treatment Government Departments have made themselves permanently responsible, and when that arrangement is unlikely to be one with special attractions to subscribers and donors, tending rather to decrease and not increase their benefactions, it is very necessary, in fixing the amount of the grant, that all essential factors should be taken into consideration.

The accounts of voluntary hospitals are prepared, as far as is possible, on a business basis, and for this purpose the Central Funds who rule the matter have at their disposal the most skilled advice available, but this advice apparently has not been called into service in respect of the capitation grant system. The accounts differ from those of a business concern in one particular, which has in the past not been of vital importance, but which will, in view of the working arrangement between the hospitals and the Ministry of Pensions and the Ministry of Health, now become an essential consideration. This is the question of what is described as capital. The capital of a voluntary hospital in most cases consists chiefly of its land, buildings and equipment, and in any case where costing is necessary, this important fact must always be borne in mind so that, if it is possible to avoid it, the capital shall not decrease. It may, of course, be argued that renewals of buildings and equipment find their proper place in the income and expenditure account. This is so to a certain extent, but to a certain extent only. For many years the fabric of a modern hospital and its equipment are to all intents and purposes kept at approximately the same value by means of the year-to-year renewals. There arrives a time, however, in the history of a hospital, when these renewals become something more than an annual expense. This is a plain fact as applied to every building, but it becomes more striking when it is applied to hospitals which were built, say, at the beginning of last century or hospitals which are entirely scrapped and rebuilt in another neighbourhood. It is evident, therefore, that in any scientific system of costing an allowance for the renewal of the fabric must find its proper place.

When fixing the amount of a capitation grant Government Departments usually employ the rough-and-ready means of an examination of the average cost of the occupied bed in the voluntary hospital concerned. Plainly this is wrong and the Government is taking advantage of the benefactions of those who in the past provided the funds to buy the land and erect and furnish the buildings which are used as hospitals. This is a reproach which it should not be possible to apply to a Government professing the wish to avoid any part of the cost it incurs falling upon the voluntary resources of a Charity.

The capitation system, therefore, on its present basis, is entirely wrong, and in the negotiations which recently took place between the British Hospitals Association and the Ministry of Pensions, a most important factor of the question was apparently overlooked. To put the matter right it will be necessary for each hospital which accepts Government patients to calculate what is the amount of its capital, *i.e.*, that represented by land, buildings and equipment, which is used for the maintenance of Government patients. The next step will be to decide what is a fair interest to charge on such capital and to translate that interest into a sum per bed per day for the purpose of adjusting the capitation grant to its proper proportions. We calculate that in respect of a hospital whose capital of this character represents £120,000 (probably that of a hospital of about 200 beds) the proper addition to the capitation-grant would be about 1s. 6d. per day. This supplement should be collected in all cases, earmarked and placed to a reserve fund, the ultimate object of which is renewal of fabric or major expenditure of the kind.

This examination of the subject leads us to another consideration, and that is the question of the payment of medical men who attend upon Government patients while in hospital. We were never greatly in love with the system of a percentage of the capitation grant being placed on one side for the purpose, because the medical man was placed in the invidious position of gaining more on a high cost than a low. When it comes, as it must inevitably do, to that percentage including an element represented by capital, *i.e.*, past benefactions, it is plain that medical men could not and would not take advantage of the hospitals' position in that respect. We sincerely hope that the British Medical Association will reconsider this question of the method of the payment of medical men (the main principle being one, of course, with which we entirely agree) and examine the much preferable system of a separate Government contribution for the purpose of their remuneration over and above the capitation grant for maintenance. As we have said in previous articles, the part of hospital revenues which is represented by payments made by patients, or made in their behalf, is certain to become a much larger proportion of total income than it has been in the past. How desirable, therefore, is it to arrange such payments on a uniform and scientific basis; it is to help to bring this about we offer the Committees of Management of the hospitals the above contribution to what must be an important part of their deliberations.

